# Pyroxasulfone and Isoproturon Nanosuspensions for Sustainable Wheat Production: Efficacy, Safety, and Yield Benefits

**DOI:** 10.3390/plants15172608

**Published:** 2026-08-26

**Authors:** Xiaolin Wang, Xiaomeng Guo, Xuebiao Zhang, Chao Xu, Dongsheng Li, Kebing Yao, Xuejian Cheng, Kang Miao

**Affiliations:** 1 Jiangsu Hilly Area Zhenjiang Institute of Agricultural Science, Jiangsu Academy of Agricultural Sciences, Zhenjiang 212400, China; morethan365@126.com (X.W.); xiaomengguo@outlook.com (X.G.);; 2 National Engineering Research Center of Pesticide, Nankai University, Tianjin 30071, China

**Keywords:** herbicide, nanosuspension, wheat field weeds, pyroxasulfone, isoproturon

## Abstract

Wheat production, vital for global food security, faces significant threats from weed competition. Conventional herbicide formulation with high application dosage raises environmental concerns, driving the need for efficient, low-dosage delivery systems such as nanoherbicides. Herein, the pyroxasulfone nanosuspension and isoproturon nanosuspension with Z-average particle sizes less than 250 nm were prepared by wet media milling. The prepared nanosuspensions exhibited excellent storage and dilution stability. Field trials showed that the pyroxasulfone and isoproturon nanosuspension significantly enhanced the herbicidal activity against *Alopecurus japonicus*, *Alopecurus aequalis*, and *Beckmannia syzigachne*, and Moreover, wheat did not exhibit noticeable symptoms of damage after being treated pyroxasulfone and isoproturon nanosuspensions, and the wheat ear count, grain number, and grain yield were significantly increased compared to the blank control. These findings provide valuable insights into economical and efficient management in wheat fields, thereby contributing to herbicide dosage and environmental risks.

## 1. Introduction

Wheat, a cornerstone of global food security, is a staple for nearly one-third of the world’s population and accounts for roughly 20% of humanity’s dietary caloric intake [1,2]. Nevertheless, the wheat yield and production stability are consistently jeopardized by weed pressure [3,4]. These pervasive biological constraints represent a major cause of crop loss, with infestations capable of reducing wheat harvests by 25–30% and posing the single largest potential threat among various yield-limiting factors, including pests and diseases [4,5]. In modern agriculture, synthetic herbicides have become an indispensable management tool due to their cost-effectiveness, high efficacy, and labor-saving advantages, playing a critical role in safeguarding yields and meeting the food demands of a growing population [6,7,8]. However, the widespread and often intensive application of these agrochemicals has triggered a series of ecological and environmental concerns [9,10]. Consequently, developing strategies to maintain effective weed control in wheat production while dramatically reducing herbicide application dosages presents a pressing and formidable challenge for sustainable agriculture.

In the past decade, nanotechnology-driven innovations in sustainable agriculture have paved entirely new pathways for the efficient delivery of herbicides [11,12,13]. Nanoherbicides, characterized by their small size and high specific surface area, can significantly improve the dispersibility, solubility, and stability of poorly soluble herbicide active ingredients while enhancing their efficiency in penetrating weed biological barriers [14,15]. For instance, Ren et al. reported that metal–organic framework (ZIF-8) nanoparticles loaded with metolachlor significantly reduced leaching and ecotoxicity and improved control efficacy against barnyardgrass [16]. It was reported that the poly(epsilon-caprolactone) nanoparticles can enhance the post-emergence herbicidal activity of atrazine against mustard plants [17]. Moreover, a recent study showed that the metal–organic framework-functionalized hollow mesoporous nanoparticles can enhance the uptake and translocation performances of quizalofop-*p*-ethyl in barnyardgrass, thereby improving the herbicidal activity [18]. In contrast, carrier-free nanosuspensions offer distinct economic and practical advantages over these carrier-based nanodelivery systems: they eliminate the need for complex carrier synthesis, avoid potential carrier-associated toxicity, achieve ultra-high active ingredient loading capacities (approaching 100%), require minimal use of organic solvents and surfactants, and involve simpler, more cost-effective production processes that are readily scalable for commercial manufacturing. Therefore, there is an urgent need to develop nanoherbicides with simple preparation processes and low costs to tackle the current challenges.

As a noncarrier-coated nanopesticide, nanosuspension offers advantages such as a high loading capacity, the absence of organic solvents, low production costs, and low surfactant usage [19,20,21]. Zhu et al. prepared an azoxystrobin nanosuspension with a particle size of 83 nm using the flash nanoprecipitation technique, and the median effective dose of the azoxystrobin nanosuspension against *Thanatephorus cucumeris* was only 40.72% of that for the commercially available formulation [22]. Corrias et al. constructed a zoxamide nanosuspension by wet media milling, and the field trials demonstrated that the nanosuspension enhanced the accumulation and retention of zoxamide in the tomato plants [23]. Ding et al. reported that the chlorantraniliprole nanosuspension showed superior dispersibility, superior foliar wetting, and retention performances, thereby further enhancing the bioavailability of chlorantraniliprole against *Cnaphalocrocis medinalis* compared to a commercial suspension concentrate [24]. Additionally, the nanosuspension was also reported to enhance the deposition, uptake, and post-emergence herbicidal activity of quinclorac [25]. Nevertheless, field data on the biological activity of nanosuspensions remain relatively scarce, and the safety of herbicide-loaded nanosuspensions for crops under field conditions requires rigorous validation.

Pyroxasulfone, an isoxazoline herbicide, can control multiple annual weeds by inhibiting the biosynthesis of very long chain fatty acids when applied pre-emergence or early post-emergence [26,27]. Isoproturon is a phenylurea-derived systemic herbicide with a complementary mode of action—it inhibits electron transport in Photosystem II (PSII) as a post-emergence herbicide and is widely used to control weeds in wheat [28,29]. The combination of pyroxasulfone (VLCFA elongase inhibitor, pre-emergence/early post-emergence) and isoproturon (PSII inhibitor, post-emergence) provides complementary and overlapping weed control spectra, which can effectively manage diverse weed populations in wheat fields and potentially enhance the wheat grain yield while mitigating the risk of herbicide resistance [30]. However, to the best of our knowledge, the efficacy of the combined application of pyroxasulfone and isoproturon for controlling weeds in wheat fields has not yet been explored. Therefore, we aimed to (1) prepare pyroxasulfone nanosuspension and isoproturon nanosuspension using wet media milling technology, (2) characterize their physical stability, (3) evaluate their field herbicidal activity, and (4) assess their effect on wheat yield.

## 2. Materials and Methods

### 2.1. Materials

Pyroxasulfone (Pyr) technical material (purity > 97%) was acquired from Shanghai Qunli Chemical Co., Ltd., (Shanghai, China). Isoproturon (Iso) technical material (purity > 97%) was obtained from Kuaida Agrochemical Co., Ltd., (Nantong, China).

Pyroxasulfone suspension concentrate (Pyr@SC) was purchased from Shanghai Qunli Chemical Co., Ltd., (Shanghai, China). Isoproturon suspension concentrate (Iso@SC) was purchased from Jiangsu Dongbao Agrochemical Co., Ltd., (Nantong, China). Dispersants GY1150 and GY1200 were provided by Grand Agrochem Co., Ltd., (Beijing, China). Dispersants Agrilan 700, Morwet D425, and Ufoxane 3A were acquired from Jierun Technology Co., Ltd., (Nanjing, China). Dispersant Atlox 4917 was obtained from Corda Crop Care (Shanghai, China). Dispersant PEAS 03 was received from Timing International Trading Co., Ltd., (Shanghai, China). The chemical structure formulas of the dispersants are presented in the Supporting Information Table S1. Silicone antifoam SAG 1572 was purchased from Jierun Technology Co., Ltd., (Nanjing, China). The wetting agent dioctyl sulfosuccinate sodium salt (AOT) was provided by Jiangsu Hai’an Petrochemical Company (Nantong, China). The wetting agent butylnaphthalenesulfonic acid sodium salt (BAS) was purchased from Shandong Yousuo Chemical Technology Co., Ltd., (Linyi, China). Antifreeze propylene glycol and thickener xanthan gum were purchased from Shanghai Macklin Biochemical Co., Ltd., (Shanghai, China). All chemicals were used as supplied.

### 2.2. Formulation of Nanosuspension

The pyroxasulfone nanosuspension (Pyr@NS) and the isoproturon nanosuspension (Iso@NS) were formulated using wet media milling, as described in the reference, with some modifications [25]. First of all, 4% dispersant, 1% wetting agent, 15.5% Pyr or Iso technical material powder, 5% antifreeze propylene glycol, 0.2% thickener xanthan gum, and 74.3% water were premixed under magnetic stirring to prepare a coarse suspension. The formulation compositions are listed in Table 1. Subsequently, the suspension was introduced into a unit of grinding equipment (WG-0.3; Suzhou Vgreen Nano-Chem Technology Co., Ltd., Suzhou, China) containing 0.3 mm zirconia beads as the milling media and processed for 120 min to prepare the nanosuspensions. The rotary speed of the grinding equipment was set at 2000 rpm. During the wet media milling process, 50 µL of silicone antifoam (approximately 0.1% *w*/*w* relative to the total batch weight) was added to the grinding vessel every 30 min using a calibrated micropipette to eliminate foam generated during grinding.

### 2.3. Measurement of Particle Size and Polydispersity Index

The Z-average particle size, polydispersity index (PDI), D_10_, D_50_, and D_90_ of Pyr@NS and Iso@NS were determined at 25 °C using a BeNano 180 zeta and particle size analyzer (BT-9300H; Dandong Bettersize Instrument Co., Ltd., Dandong, China), which is a dynamic light scattering (DLS) system. The D_50_ and D_90_ values were derived from volume-weighted distributions converted by the instrument’s software [31]. Before measurement, Pyr@NS and Iso@NS were diluted 200-fold with deionized water, and all measurements were performed in triplicate.

### 2.4. Morphological Characterization

The morphology of Pyr@NS and Iso@NS was captured using scanning electron microscopy (SEM, SU8010; Hitachi, Tokyo, Japan) at an acceleration voltage of 3 kV. Prior to platinum sputter coating under vacuum treatment, Pyr@NS and Iso@NS were respectively diluted 300 times with deionized water. Subsequently, a 5 μL droplet of each diluted sample was deposited on clean silicon wafers and air-dried at ambient temperature.

### 2.5. Accelerated Storage Stability Test

The accelerated storage stability of Pyr@NS and Iso@NS was assessed according to CIPAC MT 46 [32]. The freshly prepared Pyr@NS and Iso@NS were respectively dispensed into 20 mL capped glass containers and then stored at 54 ± 2 °C for 14 days to conduct an accelerated storage stability test. After storage, the Z-average particle size and PDI of Pyr@NS and Iso@NS were measured.

### 2.6. Dilution Stability Assay

To monitor changes in particle size after dilution, the Pyr@NS and Iso@NS were respectively diluted 200-fold with deionized water, and the Z-average particle size and PDI of the dilutions were continuously determined within 5 h using the BeNano 180 zeta and particle size analyzer at 25 °C. Meanwhile, the colloidal stability of the diluted suspensions was evaluated using a Turbiscan Lab Tower (Formulaction/Microtrac, L’Union, France). The diluted samples were introduced into the sample vials and scanned every 15 min at 25 °C for 5 h. The colloidal stability of dilutions was reflected by the changes in transmitted and backscattered light data over time. The Turbiscan Stability Index (TSI) was calculated to quantify variations in backscattered light data. Specifically, TSI was derived using Equation (1):

(1) $$\begin{array}{c} TSI=\sqrt{\frac{\sum_{i=1}^{n}(x_{i}-x_{BS}{)}^{2}}{n-1}} \end{array}$$

where n denotes the number of scans, $x_{i}$ represents the average backscattering for each minute of measurement, and $x_{BS}$ represents the average $x_{i}$.

### 2.7. Content Analysis

The content of Pyr was determined using Dionex Ultimate 3000 (Thermo Fisher Scientific, Waltham, MA, USA) high-performance liquid chromatography (HPLC) equipped with an Eclipse XDB-C18 column (5 μm, 4.6 × 250 mm) at 30 °C. The mobile phases were composed of acetonitrile and 0.1% phosphoric acid aqueous solution (50:50, *v*/*v*). The wavelength of the UV detector was set to 226 nm. For Iso, the chromatographic column was a Zorbax SB-C18 column (5 μm, 4.6 × 250 mm), and the mobile phases consisted of methanol and water (70:30, *v*/*v*). The UV detector wavelength was 280 nm. The syringe volume for all samples was 10 µL, and the flow rate of mobile phases was 1.0 mL min^−1^.

### 2.8. Field Herbicidal Activity Assessment

The herbicidal activity of Pyr@NS and Iso@NS was assessed in wheat fields from January 2025 to April 2025 in Zhenjiang City, Jiangsu Province, China (31.9646° N, 119.3111° E). The herbicides were applied about 7 weeks after the wheat was sown, when the wheat seedlings had uniformly emerged. Spray treatments were applied in the field using a knapsack sprayer operated at a pressure of 0.30–0.45 MPa and a flow rate of 1.5 L min^−1^ (3WBS-D-16B; Zhengzhou Xinxiu Agricultural Machinery Co., Ltd., Zhengzhou, China), equipped with a fan nozzle (F100-03; Happiness Electric Appliance Factory, Taizhou, China). The spray volume was set at 450 L ha^−1^. The applied dosages and the formulation of herbicides are listed in Table 2. Each treatment was carried out on a 48 m^2^ wheat plot with four replicates, and each wheat plot was randomly arranged. The dominant weed species in the wheat fields were *Alopecurus japonicus*, *Alopecurus aequalis*, and *Beckmannia syzigachne*. Crop safety was visually assessed 30 days post-application to evaluate wheat injury. After 30 days and 104 days of treatment, the number of weed plants in each treatment plot was counted by randomly selecting four areas of 0.25 m^2^, and the fresh weight of weeds at each sampling point was assessed after 104 days of treatment. The weed control efficacy and the fresh weight control effect can be calculated according to Equations (2) and (3).

(2) $$\mathrm{Weed}\mathrm{control}\mathrm{efficacy}\left(\%\right)=\frac{\mathrm{number}\mathrm{of}\mathrm{weed}\mathrm{plants}\mathrm{of}\mathrm{CK}-\mathrm{number}\mathrm{of}\mathrm{weed}\mathrm{plants}\mathrm{of}\mathrm{treatment}}{\mathrm{number}\mathrm{of}\mathrm{weed}\mathrm{plants}\mathrm{of}\mathrm{CK}}$$

(3) $$\mathrm{Fresh}\mathrm{weight}\mathrm{control}\mathrm{effect}\left(\%\right)=\frac{\mathrm{the}\mathrm{fresh}\mathrm{weight}\mathrm{of}\mathrm{CK}-\mathrm{the}\mathrm{fresh}\mathrm{weight}\mathrm{of}\mathrm{treatment}}{\mathrm{the}\mathrm{fresh}\mathrm{weight}\mathrm{of}\mathrm{CK}}$$

After the wheat harvest, the number of ears in each treatment plot was counted by randomly selected 1 m^2^ quadrats. Forty ears of wheat were randomly selected from each plot as samples for measuring the grain number. The selected wheat was dried at 65 °C and threshed, after which the thousand-grain (TGM) weight was measured. The wheat yield was calculated according to Equation (4).

(4) $$Wheat\;yield\;\left({\mathrm{kg}\mathrm{ha}}^{-1}\right)=\frac{ear\;count\;\left(ear\;m^{-2}\right)\times grain\;number\;\left(grains\;ear^{-1}\right)\times TGW}{100}$$

### 2.9. Statistical Analysis

The data were displayed as the mean ± standard deviation and analyzed with Duncan’s multiple range test at a significance level of 0.05 with statistical analysis software DPS (version 7.05).

## 3. Results and Discussion

### 3.1. Formulation Optimization

**Figure 1:**
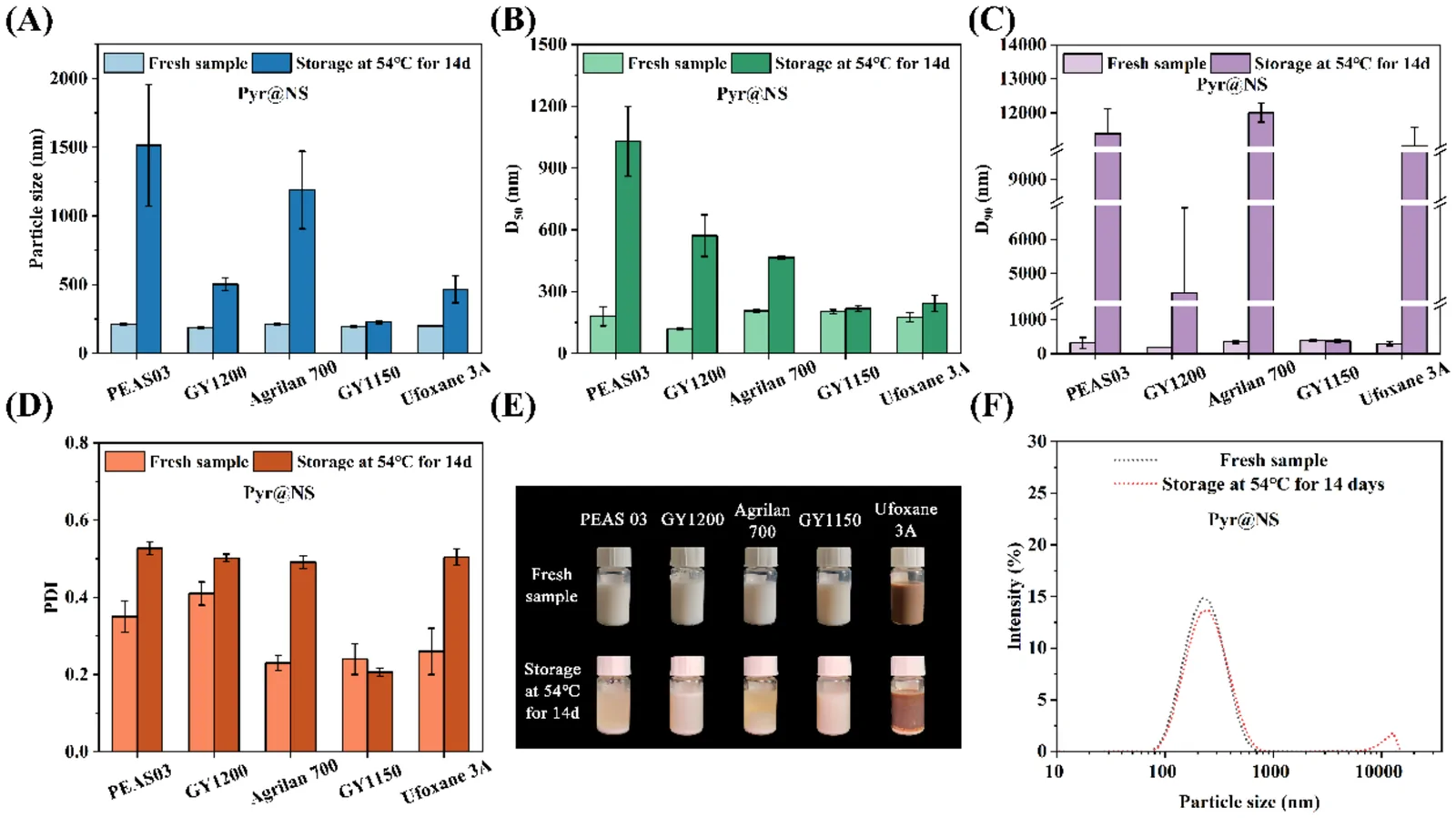
Formulation optimization of Pyr@NS: (**A**) variation in Z-average particle size after accelerated storage, (**B**) variation in D50 after accelerated storage, (**C**) variation in D90 after accelerated storage, (**D**) variation in PDI after accelerated storage, (**E**) appearance change after accelerated storage, (**F**) particle size distribution of Pyr@NS prepared using GY1150 as the dispersant after accelerated storage.

The appropriate dispersant is crucial for the physical stability of nanosuspensions, as it is adsorbed onto the surface of pesticide nanoparticles to inhibit particle growth [33,34]. Thus, different types of dispersants were evaluated. According to the data in Figure 1A, the Z-average particle sizes of fresh Pyr@NS prepared by five different dispersants were consistently below 250 nm, which indicated that the selected five dispersants effectively promoted the formation of Pyr@NS. After storage at 54 °C for 14 days, the Z-average particle sizes of Pyr@NS prepared employing PEAS03, GY1200, Agrilan 700, and Ufoxane 3A as dispersant were significantly increased to 1515.28 nm, 503.41 nm, 1189.69 nm, and 465.34 nm. However, when the dispersant was GY1150, the Z-average particle size slightly increased from 195.41 nm to 226.71 nm after storage at 54 °C for 14 days. Except for PEAS03, the D_50_ of Pyr@NS prepared with the other four dispersants was less than 1000 nm after storage at 54 °C for 14 days (Figure 1B), indicating that over 50% of the Pyr particles remained at the nanoscale. Moreover, Figure 1C showed that the D_90_ of Pyr@NS prepared using PEAS03, GY1200, Agrilan 700, and Ufoxane 3A as dispersants increased to 11,391.22 nm, 4428.1 nm, 12,005.51 nm, and 11,030.67 nm, respectively. This increase can be attributed to the poor affinity between the four types of dispersants and Pyr particles. Notably, the D_90_ of Pyr@NS prepared by using GY1150 as a dispersant hardly changed after storage at 54 °C for 14 days. In addition, the PDI exhibited a marked increase after storage at 54 °C for 14 days when the dispersant was PEAS03, GY1200, Agrilan 700, and Ufoxane 3A, whereas the PDI of Pyr@NS prepared with the dispersant GY1150 showed no substantial variations (Figure 1D). The low PDI values imply a uniform particle size distribution of the dispersion system [35]. Therefore, the above results illustrate that, compared to the other four dispersants, GY1150 can more effectively stabilize Pyr@NS. Furthermore, the appearance change before and after accelerated storage revealed insights into the stability of Pyr@NS (Figure 1E). The appearance of Pyr@NS prepared by GY1150 remained uniformly dispersed even after storage at 54 °C for 14 days, as the Brownian motion of Pyr nanoparticles can overcome gravity-driven sedimentation. However, the Pyr@NS prepared with the other four dispersants showed apparent phase separation after storage at 54 °C for 14 days, which can be attributed to sedimentation caused by particle growth. Figure 1F indicated that the particle size distribution of Pyr@NS prepared using GY1150 as a dispersant hardly changed after storage at 54 °C for 14 days, which further demonstrated the excellent dispersing performance of GY1150 for Pyr.

**Figure 2:**
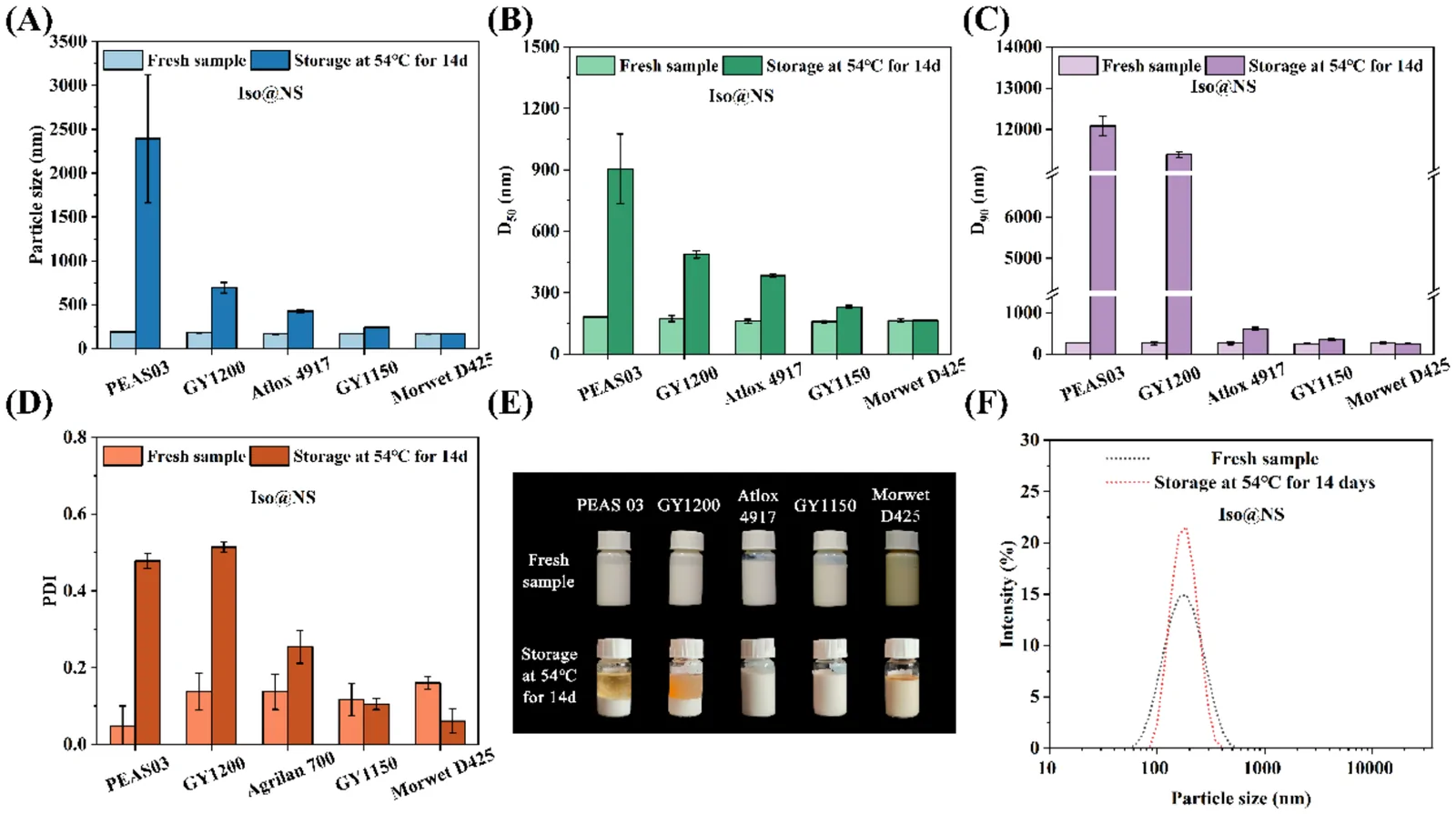
Formulation optimization of Iso@NS: (**A**) variation in Z-average particle size after accelerated storage, (**B**) variation in D50 after accelerated storage, (**C**) variation in D90 after accelerated storage, (**D**) variation in PDI after accelerated storage, (**E**) appearance change after accelerated storage, (**F**) particle size distribution of Iso@NS prepared using Morwet D425 as the dispersant after accelerated storage.

Figure 2 displays the formulation optimization results of Iso@NS. As can be seen in Figure 2A, the Z-average particle sizes of Iso@NS were smaller than 500 nm after storage at 54 °C for 14 days when the dispersant species were Atlox 4917, GY1150, and Morwet D425, while the Z-average particle sizes of Iso@NS prepared using PEAS03 and GY1200 as dispersants increased to more than 500 nm after storage at 54 °C for 14 days. Similarly, the D_50_, D_90_, and PDI of Iso@NS did not undergo a dramatic increase after storage at 54 °C for 14 days when the dispersant species were Atlox 4917, GY1150, and Morwet D425, whereas the D_50_, D_90_, and PDI of Iso@NS significantly increased after storage at 54 °C for 14 days when the dispersant was PEAS03 and GY1200 (Figure 2B–D). Furthermore, due to the dramatic particle growth, the Iso@NS prepared with the dispersants PEAS03 and GY1200 showed apparent delamination between the Iso particles and the aqueous phase after storage at 54 °C for 14 days (Figure 2E). Conversely, the Iso@NS prepared by Atlox 4917, GY1150, and Morwet D425 as dispersants maintained uniform dispersion after storage at 54 °C for 14 days. Notably, the dispersant Morwet D425 displayed the superior dispersing performance for Iso, as the Iso@NS prepared by Morwet D425 exhibited the smallest D_50_, D_90_, and PDI after storage at 54 °C for 14 days. Moreover, the particle size distribution of Iso@NS prepared using Morwet D425 as a dispersant did not show significant broadening (Figure 2F). Therefore, these results indicate that Morwet D425 was an appropriate dispersant for Iso. All subsequent experiments were conducted using the optimized formulations Pyr@NS and Iso@NS, with the specific composition details listed in Table 1.

### 3.2. Morphology of Pyr@NS and Iso@NS

**Figure 3:**
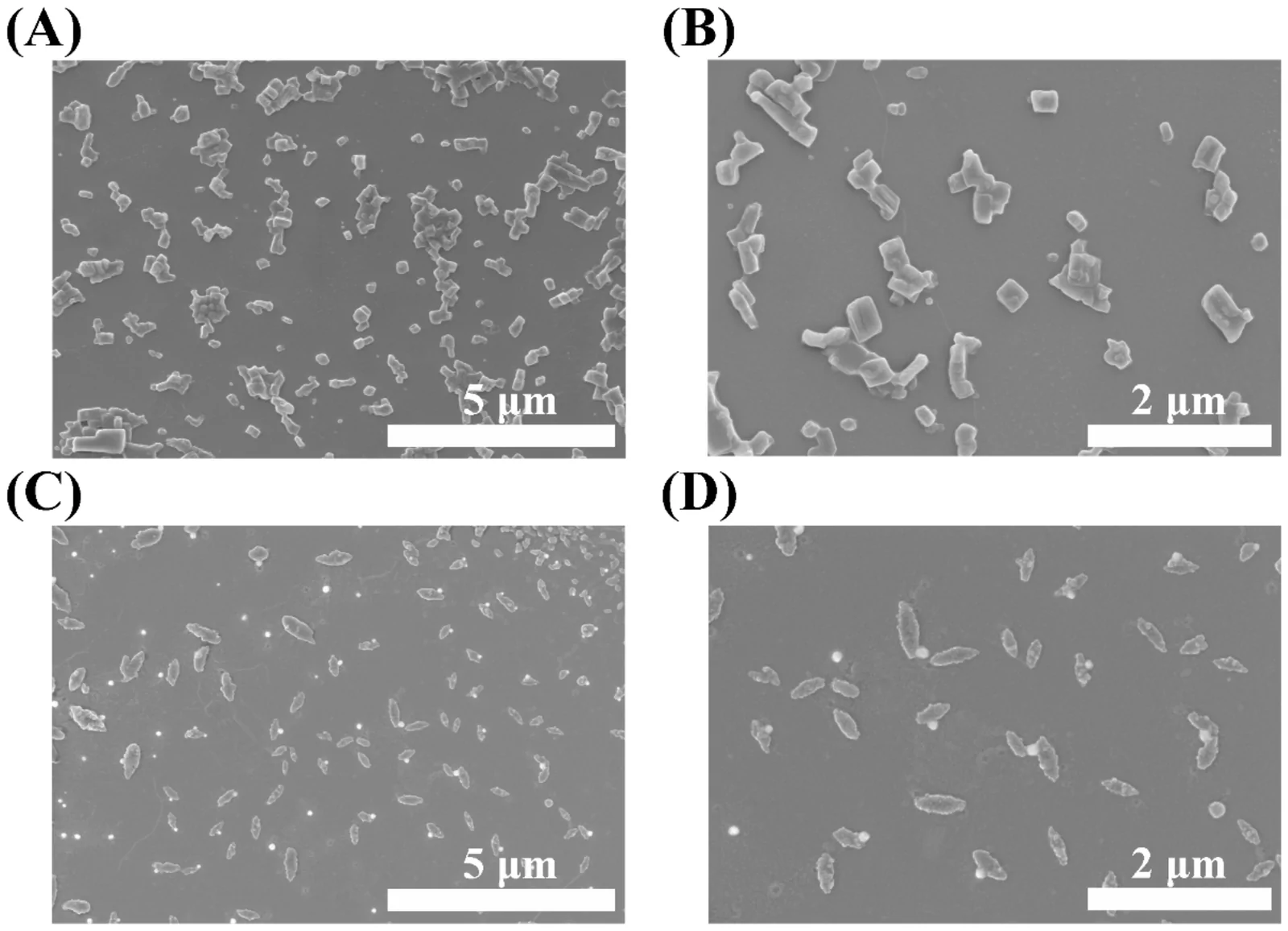
Scanning electron microscopy images of Pyr@NS and Iso@NS at different magnifications: (**A**) Pyr@NS at 10 k, (**B**) Pyr@NS at 20 k, (**C**) Iso@NS at 10 k, and (**D**) Iso@NS at 20 k.

The morphology of Pyr@NS and Iso@NS was characterized by SEM. Figure 3A,B showed that the Pyr nanoparticles in the Pyr@NS exhibited a sharply defined polyhedral shape. However, the Iso nanoparticles in the Iso@NS exhibited irregular spindle shapes with a high aspect ratio (Figure 3C,D). The morphological differences between Pyr nanoparticles and Iso nanoparticles may be attributed to the distinct crystal structures of the technical materials. Furthermore, these results confirmed that Pyr nanoparticles and Iso nanoparticles were monodisperse and maintained at the nanoscale after water evaporation.

### 3.3. Dilution Stability

**Figure 4:**
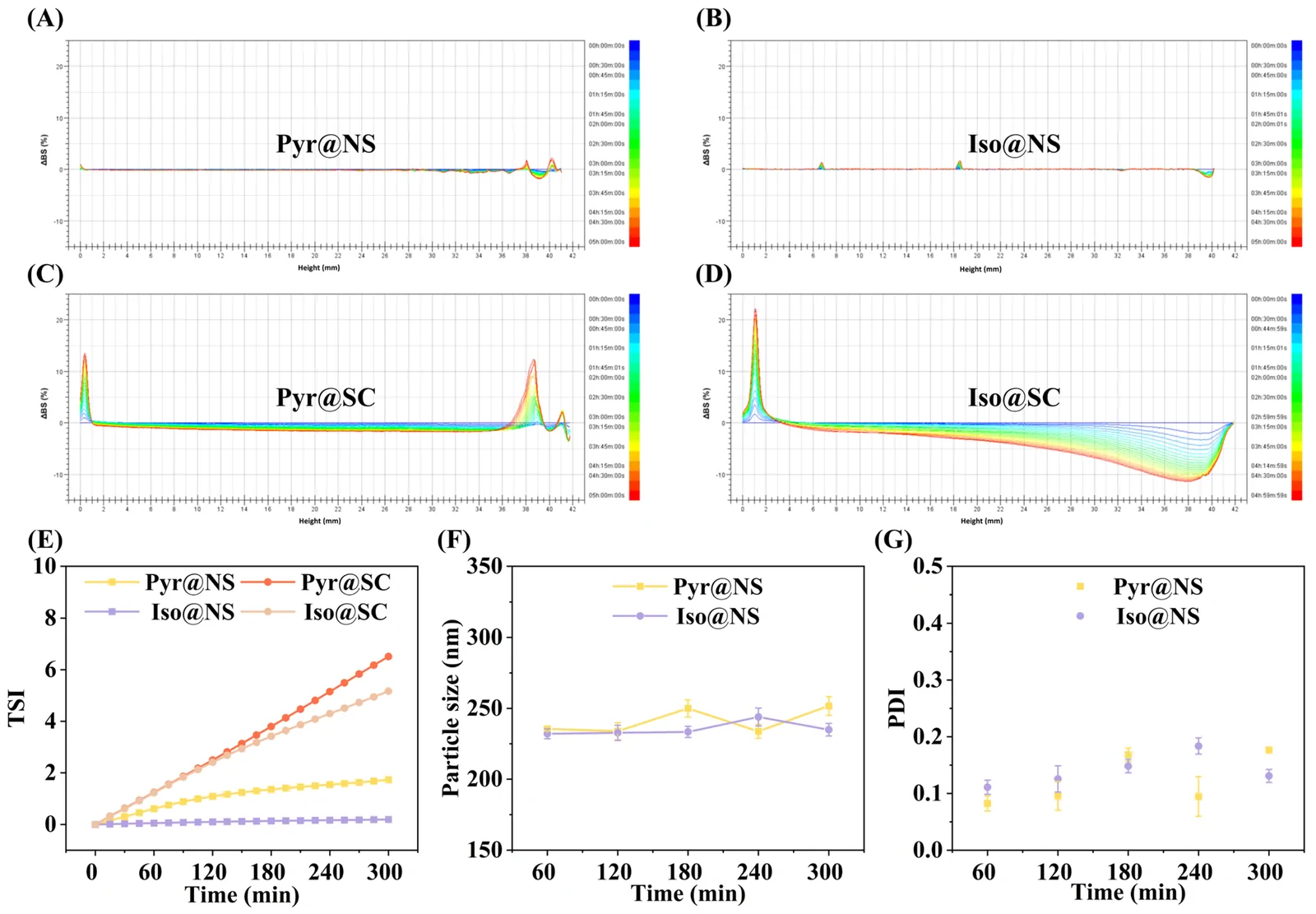
Dilution stability of Pyr@NS and Iso@NS: (**A**) backscattering profiles of Pyr@NS, (**B**) backscattering profiles of Iso@NS, (**C**) backscattering profiles of Pyr@SC, (**D**) backscattering profiles of Iso@SC, (**E**) TSI values, (**F**) variation of particle size in 5 h, and (**G**) variation of PDI in 5 h.

To verify the physical stability of Pyr@NS and Iso@NS after dilution with water, the Z-average particle size and colloidal stability of the dilutions were continuously monitored over 5 h. Figure 4A–D illustrate the backscattered spectra of the dilutions of the four formulations, respectively, which reflect the colloidal stability of the dilutions. Specifically, the horizontal axis scale of the spectra corresponds to different heights of the dilutions, and the vertical axis scale of the spectra represents the intensity of backscattered light. The different colors correspond to backscattered spectra at different scan times. It can be clearly seen in Figure 4A,B that the backscattered light intensity of Pyr@NS and Iso@NS only exhibited slight changes over the 5 h dilution period. However, the backscattered light intensity of Pyr@SC and Iso@SC at the bottom increased, which can be attributed to the increased particle concentration at the bottom (Figure 4C,D). Moreover, the backscattered light intensity at the top of Pyr@SC and Iso@SC also exhibited a distinct increase or decrease (Figure 4C,D), which implied that the dispersion system becomes increasingly non-uniform over time. The data in Figure 4E show that the TSI values of Pyr@NS and Iso@NS were significantly lower than those of Pyr@SC and Iso@SC. The lower TSI values of the nanosuspensions can be explained by their much smaller particle sizes (below 250 nm), which lead to much slower sedimentation according to Stokes’ law, as well as the Brownian motion that effectively counteracts gravitational sedimentation. These results indicated that the dilutions of nanosuspensions demonstrated superior colloidal stability compared to the conventional formulation, as the lower TSI values indicated better colloidal stability [36]. Furthermore, the Z-average particle sizes of Pyr@NS and Iso@NS varied within the range of 220 nm to 260 nm (Figure 4F), which suggested that the Pyr nanoparticles and Iso nanoparticles hardly suffer noticeable aggregation or Ostwald ripening during the 5 h. The PDI of Pyr@NS and Iso@NS remained below 0.2 within the 5 h dilution period (Figure 4G), further demonstrating the exceptional dispersion stability of dilutions.

### 3.4. Filed Herbicidal Activity

**Figure 5:**
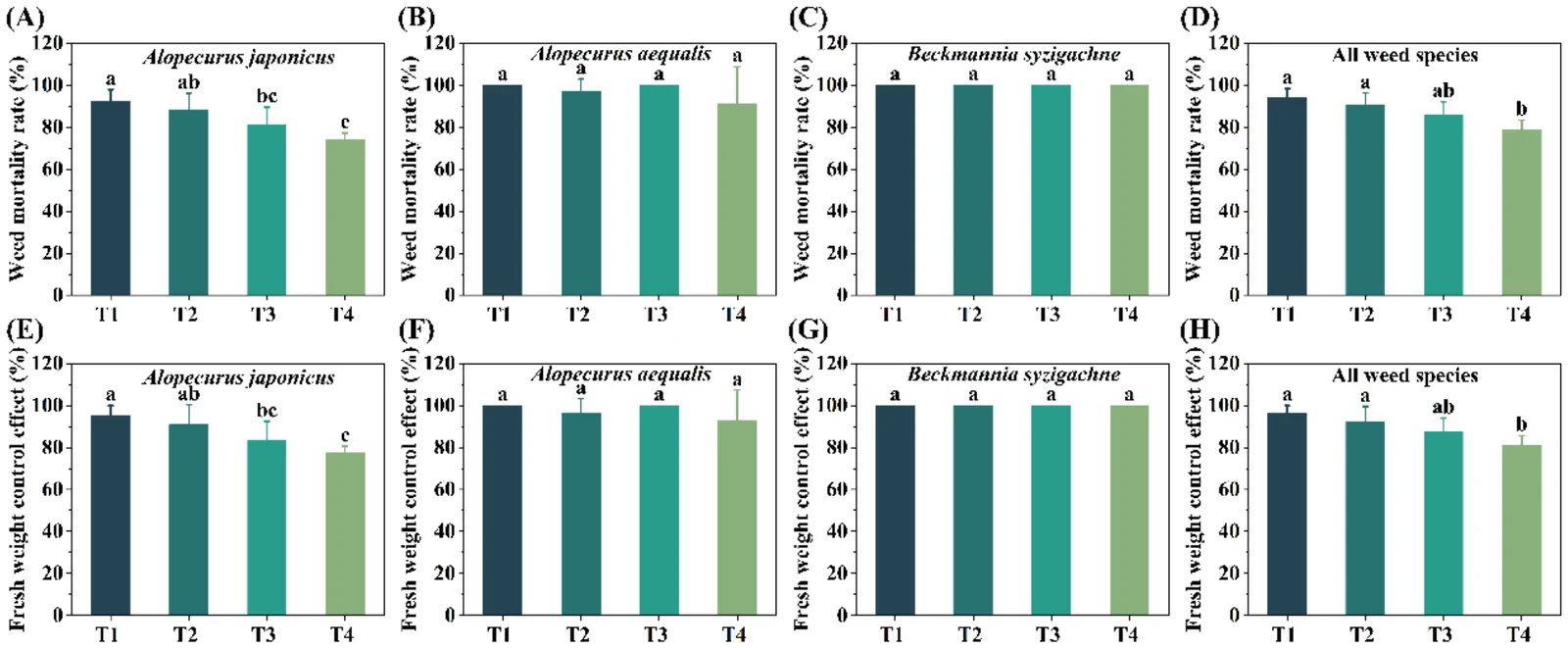
Field herbicidal activity of Pyr@NS and Iso@NS: weed after 104 days against (**A**) Alopecurus japonicus, (**B**) Alopecurus aequalis, (**C**) Beckmannia syzigachne, and (**D**) all weed species; fresh weight control effect after 104 days against (**E**) Alopecurus japonicus, (**F**) Alopecurus aequalis, (**G**) Beckmannia syzigachne, and (**H**) all weed species. Different letters above bars indicate a statistical difference at *p* < 0.05.

The weed control efficacy of the different treatments was evaluated after spraying herbicides for 30 days. The weed control efficacy of Pyr@NS and Iso@NS against *Alopecurus japonicus* was higher than that of Pyr@SC and Iso@SC after 104 days of treatment (Figure 5A). The weed control efficacy of different treatments against *Alopecurus aequalis* and *Beckmannia syzigachne* showed no significant difference (Figure 5B,C), which may be attributed to the high sensitivity of these two weeds to Pyr and Iso. The weed control efficacy of T3 against all weed species was higher than that of T4 at 104 days post-application. As shown in Figure 5A, the weed control efficacy of T1, T2, T3, and T4 against *Alopecurus japonicus*, *Alopecurus aequalis*, and *Beckmannia syzigachne* was 91.98%, 89.15%, 83.02%, and 76.42%, respectively. Notably, the dosages of Pyr and Iso of T3 were lower than that of T2, whereas no statistically significant difference in the weed control efficacy was observed between T2 and T3. These results indicate that Pyr@NS and Iso@NS exhibited superior field herbicidal activity compared to Pyr@SC and Iso@SC. Moreover, visual inspection results indicate that none of the treated wheat exhibited obvious symptoms of damage. Furthermore, the data in Figure 5E showed that the fresh weight control effect of T3 against *Alopecurus japonicus* was higher than that of T4, and the fresh weight control effect of T1 against *Alopecurus japonicus* was also higher than that of T2. Nevertheless, the difference in the fresh weight control effect of different treatments against *Alopecurus aequalis*, and *Beckmannia syzigachne* was not significant after 104 days of treatment (Figure 5F,G). The fresh weight control effect of T1, T2, T3, and T4 against all weed species was 96.17%, 92.41%, 87.34%, and 81.33%, respectively (Figure 5H). It is noteworthy that the difference in the fresh weight control effect between T2 and T3 was not significant, although the overall herbicide application dosage of T3 was 23.91% lower than that of T2. The results showed that the field herbicidal activity of Pyr@NS and Iso@NS was higher than that of Pyr@SC and Iso@SC. Moreover, the Pyr@NS and Iso@NS can reduce the herbicide application dosages in field applications, which contributes to mitigating environmental risks associated with herbicide application at the source.

### 3.5. Wheat Yield

**Figure 6:**
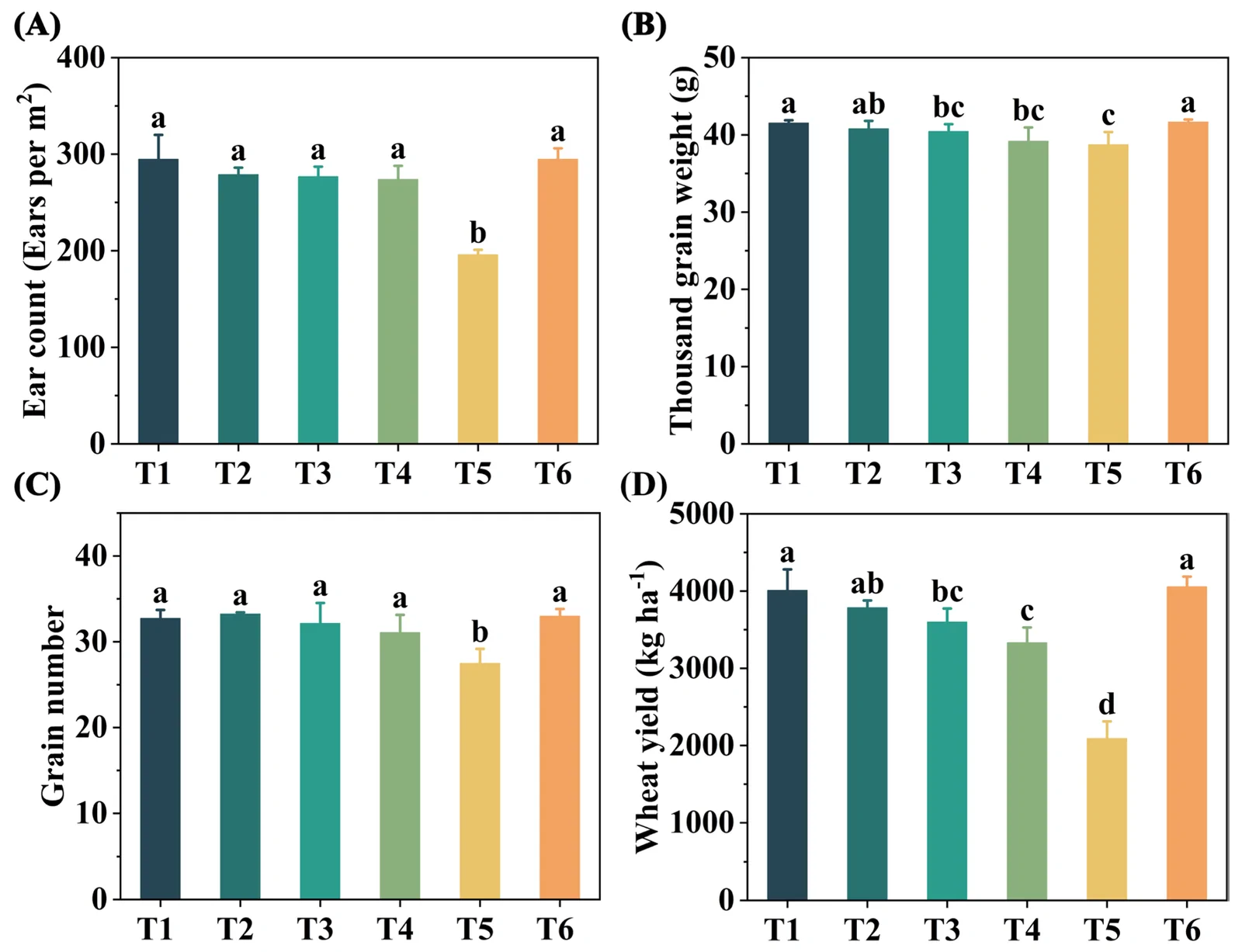
Effect of different treatments on the yield of wheat: (**A**) ear count, (**B**) thousand grain weight, (**C**) grain number, and (**D**) wheat yield. Different letters above bars indicate a statistical difference at *p* < 0.05.

The wheat yield was assessed by determining the ear count, thousand grain weight, and grain number. As shown in Figure 6A, the ear count of T1, T2, T3, T4, and T6 was significantly higher than that of T5. Moreover, the thousand grain weights of T1, T2, T3, T4, T5, and T6 were 41.55 g, 40.80 g, 40.47 g, 39.20 g, 38.74 g, and 41.68 g, and the thousand grain weights of T1 and T6 were significantly higher than those of T3, T4, and T5 (Figure 6B). These results imply that the application of Pyr@NS and Iso@NS at optimal dosages or manual weeding can substantially enhance the thousand grain weights of wheat. Furthermore, the data in Figure 6C show that the grain numbers for T1, T2, T3, T4, and T6 were significantly greater than that for T5, which indicates that spraying herbicide or manual weeding effectively reduced the negative impact of weeds on wheat grain numbers. As illustrated in Figure 6D, the wheat yield of different treatments was 4011 kg ha^−1^ (T1), 3785 kg ha^−1^ (T2), 3601 kg ha^−1^ (T3), 3334 kg ha^−1^ (T4), 2092.79 kg ha^−1^ (T5), and 4058 kg ha^−1^ (T6). Obviously, the wheat yield of T5 was significantly lower than that of the other treatments, suggesting that spraying herbicide or manual weeding was an effective strategy for minimizing wheat yield losses. Notably, despite the overall herbicide application dosage of T3 being lower than that of T2, the difference in wheat yield between T2 and T3 was not significant (Figure 6D). This can be attributed to the improved herbicidal activity of Pyr@NS and Iso@NS compared to Pyr@SC and Iso@SC, which effectively suppresses competition between weeds and wheat for essential resources. Taken together, Pyr@NS and Iso@NS can ensure wheat yields while reducing the application dosage of Pyr and Iso, which provide valuable insights for the efficient and economical management of weeds in wheat fields.

## 4. Conclusions

In conclusion, Pyr@NS and Iso@NS were prepared via wet media milling by formulation optimization. Both Pyr@NS and Iso@NS, with an Z-average particle size less than 250 nm, exhibited remarkable dispersion and physical stability and maintained nanoscale even after accelerated storage. Moreover, Pyr@NS and Iso@NS showed excellent dilution stability compared to Pyr@SC and Iso@SC, with no significant increase in particle size after dilution. Essentially, Pyr@NS and Iso@NS significantly enhanced the herbicidal activity against *Alopecurus japonicus*, *Alopecurus aequalis*, and *Beckmannia syzigachne* in the wheat field and reliably safeguarded wheat yields. These findings provide an economically viable strategy for efficient and environmentally friendly weed management strategies in wheat fields, thereby contributing to reduced herbicide dosages and environmental risks while boosting wheat yields.

## Figures and Tables

**Table 1:** The formulation compositions of Pyr@NS and Iso@NS.

Component	Pyr@NS	Iso@NS	Proportion (%)
Technical material	Pyroxasulfone	Isoproturon	15.5
Dispersant	GY1150	Morwet D425	4
Wetting agent	BAS	AOT	1
Antifreeze	Propylene glycol	Propylene glycol	5
Thickener	Xanthan gum	Xanthan gum	0.2
Dispersion medium	Water	Water	74.3

**Table 2:** The applied dosages and formulation of herbicides in the field trials.

Treatment	Formulation	Dosages (g a.i. ha^−1^)
T1	15% Pyr@NS and 15% Iso@NS	135 and 900
T2	40% Pyr@SC and 50% Iso@SC	135 and 900
T3	15% Pyr@NS and 15% Iso@NS	112.5 and 675
T4	40% Pyr@SC and 50% Iso@SC	112.5 and 675
T5	Water	0
T6	Manual weeding	0

## Data Availability

The original contributions presented in this study are included in the article/Supplementary Materials. Further inquiries can be directed to the corresponding authors.

## Supplementary Materials

The following supporting information can be downloaded at: https://www.mdpi.com/article/10.3390/plants15172608/s1. Table S1: Chemical formula of dispersants.
