# Supplementary material for: Pyroxasulfone and Isoproturon Nanosuspensions for Sustainable Wheat Production: Efficacy, Safety, and Yield Benefits
Source: Plants (Basel). 2026 Aug 26;15(17):2608. doi: 10.3390/plants15172608 (PMC13566942; doi:10.3390/plants15172608)

Pyroxasulfone and Isoproturon Nanosuspensions for Sustainable Wheat Production: Efficacy, Safety, and Yield Benefits

Xiaolin Wang [1], Xiaomeng Guo [1], Xuebiao Zhang [1], Chao Xu [1], Dongsheng Li [1], Kebing Yao [1], Xuejian Cheng [2,*] and Kang Miao [1,*]

1 Jiangsu Hilly Area Zhenjiang Institute of Agricultural Science, Jiangsu Academy of Agricultural Sciences, Zhenjiang 212400, China
2 National Engineering Research Center of Pesticide, Nankai University, Tianjin 30071, China

* Correspondence: xuejian_cheng@163.com (X.C.); miaokang@163.com (K.M.)

Table S1. Chemical formula of dispersants.

Dispersant	Chemical structure formula
GY1200	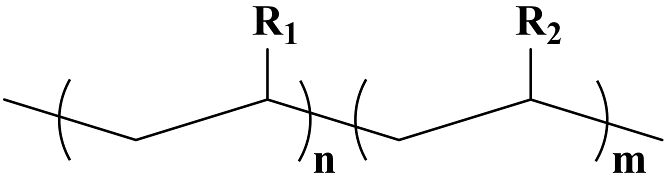
GY1150	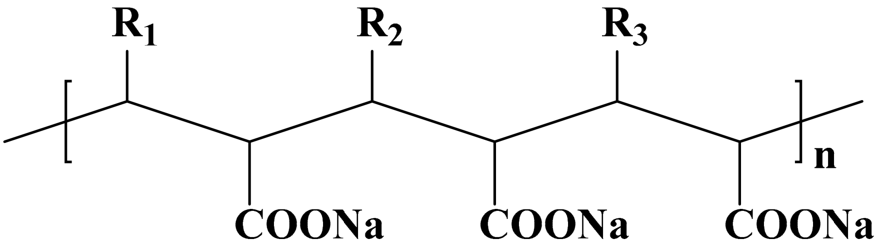
PEAS 03	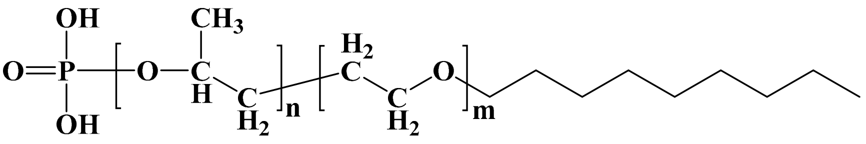
Atlox 4917	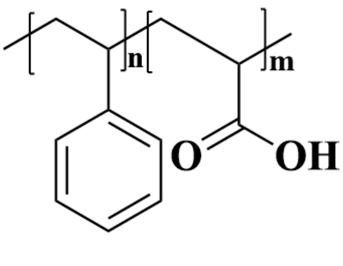

Morwet

D425

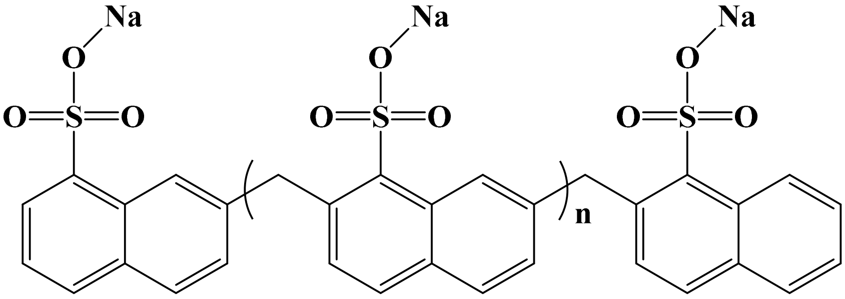

Agrilan

700

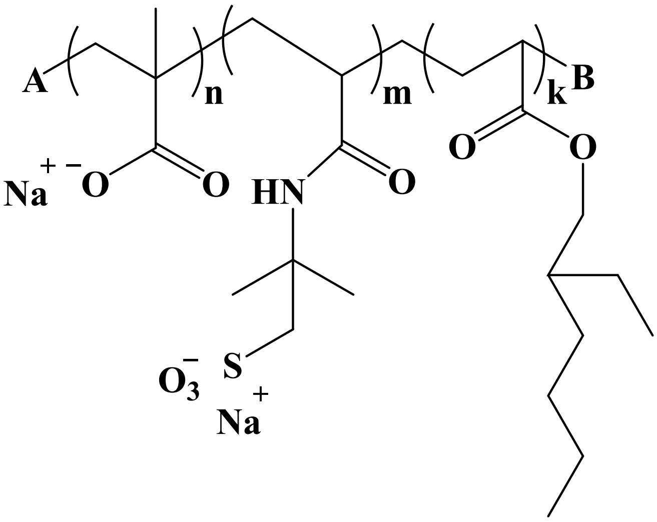

Supplement: Supplementary file 1 [file plants-15-02608-s001.zip › plants-4483287-supplementary.pdf]
